# Supplementary material for: In Vitro Resistance against DNA Gyrase Inhibitor SPR719 in Mycobacterium avium and Mycobacterium abscessus
Source: Microbiol Spectr. 2022 Jan 12;10(1):e01321-21. doi: 10.1128/spectrum.01321-21 (PMC8754139; doi:10.1128/spectrum.01321-21)
Supplement: SUPPLEMENTAL FILE 1 — Supplemental material. Download SPECTRUM01321-21_Supp_1_seq2.pdf, PDF file, 0.4 MB [file spectrum01321-21_supp_1_seq2.pdf]

SUPPLEMENTAL MATERIALS

In vitro resistance against DNA gyrase inhibitor SPR719 in *Mycobacterium avium* and *Mycobacterium abscessus*

Wassihun Wedajo Aragaw, Nicole Cotroneo, Suzanne Stokes, Michael Pucci, Ian Critchley, Martin Gengenbacher, Thomas Dick

SUPPLEMENTAL TABLES

TABLE S1. DNA sequence polymorphisms in SPR719 resistant *M. abscessus* subsp. *abscessus* ATCC19977 strains detected by whole genome sequencing.

**Exp.**	**Strain**	**DNA sequence polymorphism[#] (gene name, polymorphism)**
	wt	wt
1	Spr^{r}-L1.1	ND
	Spr^{r}-L1.2	**MAB_0006 (*gyrB*): C506A/T169N (ms)**; MAB_2374c: G64A/A22T (ms); MAB_3501: G64A/G22R (ms); MAB_4797: C230A/T77N (ms)
	Spr^{r}-S1.1	**MAB_4384: Ins247AC (fs)**; MAB_2374c: G64A/A22T (ms); MAB_2765: Del1430C (fs); MAB_3501: G64A/G22R (ms); MAB_4797: C230A/T77N (ms)
	Spr^{r}-S1.2	**MAB_4384: Ins281(sc)**; MAB_2374c: G64A/A22T (ms); MAB_2765: Del1430C (fs); MAB_3501: G64A/G22R (ms); MAB_4797: C230A/T77N (ms)
2	Spr^{r}-L2.1	**MAB_0006 (*gyrB*): C506A/T169N (ms)**; MAB_0256c: Ins181T (fs)
	Spr^{r}-L2.2	ND
	Spr^{r}-S2.1	**MAB_4384: Del273_310 (fs)**; MAB_3082: G277T/A93S (ms)
	Spr^{r}-S2.2	**MAB_4384: Ins514A (fs)**; MAB_3082: G277T/A93S (ms)
3	Spr^{r}-L3.1	ND
	Spr^{r}-L3.2	**MAB_0006 (*gyrB*): C506A/T169N (ms)**
	Spr^{r}-S3.1	**MAB_4384: Ins31C (fs)**; MAB_2374c: G64A/A22T (ms); MAB_2765: Del1430C (fs); MAB_3501: G64A/G22R (ms); MAB_4797: C230A/T77N (ms)
	Spr^{r}-S3.2	**MAB_4384: Ins31C (fs)**; MAB_2374c: G64A/A22T (ms); MAB_2765: Del1430C (fs); MAB_3501: G64A/G22R (ms); MAB_4797: C230A/T77N (ms)

Exp., independently grown culture batches; wt, wild type; Spr^{r}, SPR719 resistant strain; L, S, large and small colony size phenotype observed on SPR719 containing agar; ND, not determined. Polymorphisms in *gyrB* and MAB_4384 shown in Table 2 are highlighted in bold. Additional polymorphisms are shown in normal font. Note that several identical polymorphisms were observed in strains derived from culture batch 1 and 3, but not batch 2. The relevance of these mutations, if any, remains to be determined. [#] ms, missense mutation; Ins, insertion; Del, deletion; fs, frameshift; sc, stop codon. See Table 2 for MICs of SPR719 for the various strains.

TABLE S2. Primers used for targeted sequencing of *gyrA* and *gyrB* in *M. avium* subsp. *hominissuis* 109 and *M. abscessus* subsp. *abscessus* ATCC19977.

**Primer**	**Sequence (5'→3')**	**Target gene**	**Purpose**
***M. avium* subsp. *hominissuis* 109**			
gyrA_F1(Mav)	GATCAACAAGGACGACGGCATC	*gyrA*	Sequencing, PCR
gyrA_R1(Mav)	CAGTGGCAGTCGCGTTATGTG	*gyrA*	Sequencing, PCR
gyrA_F2(Mav)	GACGAGGAGACAGTCGATTTC	*gyrA*	Sequencing
gyrA_R2(Mav)	GTCCTCATAGCTGCGGATCTG	*gyrA*	Sequencing
gyrB_F1(Mav)	GTAGTGACCTCGCTGAAGATC	*gyrB*	Sequencing, PCR
gyrB_F2(Mav)	AGAACAGCGGCTACAACGTC	*gyrB*	Sequencing
gyrB_F3(Mav)	GTGATCTCGGTCAAGGTGAG	*gyrB*	Sequencing
gyrB_R1(Mav)	CATCGCGTAATCGATGTAGCTG	*gyrB*	PCR
***M. abscessus* subsp. *abscessus* ATCC 19977**			
gyrA_F1(Mab)	GCATCTAAAGCCGCTGAGAACG	*gyrA*	Sequencing, PCR
gyrA_F2(Mab)	GCGGGCATCTCCAACATCGAGG	*gyrA*	Sequencing
gyrA_R1(Mab)	GAGGTTGTTCAGCACCACCTTGG	*gyrA*	Sequencing
gyrA_R2(Mab)	GGTCCACGGGGCGTTCGTTTGC	*gyrA*	Sequencing, PCR
gyrB_F1(Mab)	GGCGTGGTGACGAGTTTAAAG	*gyrB*	Sequencing, PCR
gyrB_F2(Mab)	GAGATCTTCGAGACCACCACCTA	*gyrB*	Sequencing
gyrB_F3(Mab)	GCAAGAGTGCCACCGATATC	*gyrB*	Sequencing
gyrB_R1(Mab)	GTAAGTACGACGGCACAACG	*gyrB*	PCR

SUPPLEMENTAL FIGURES

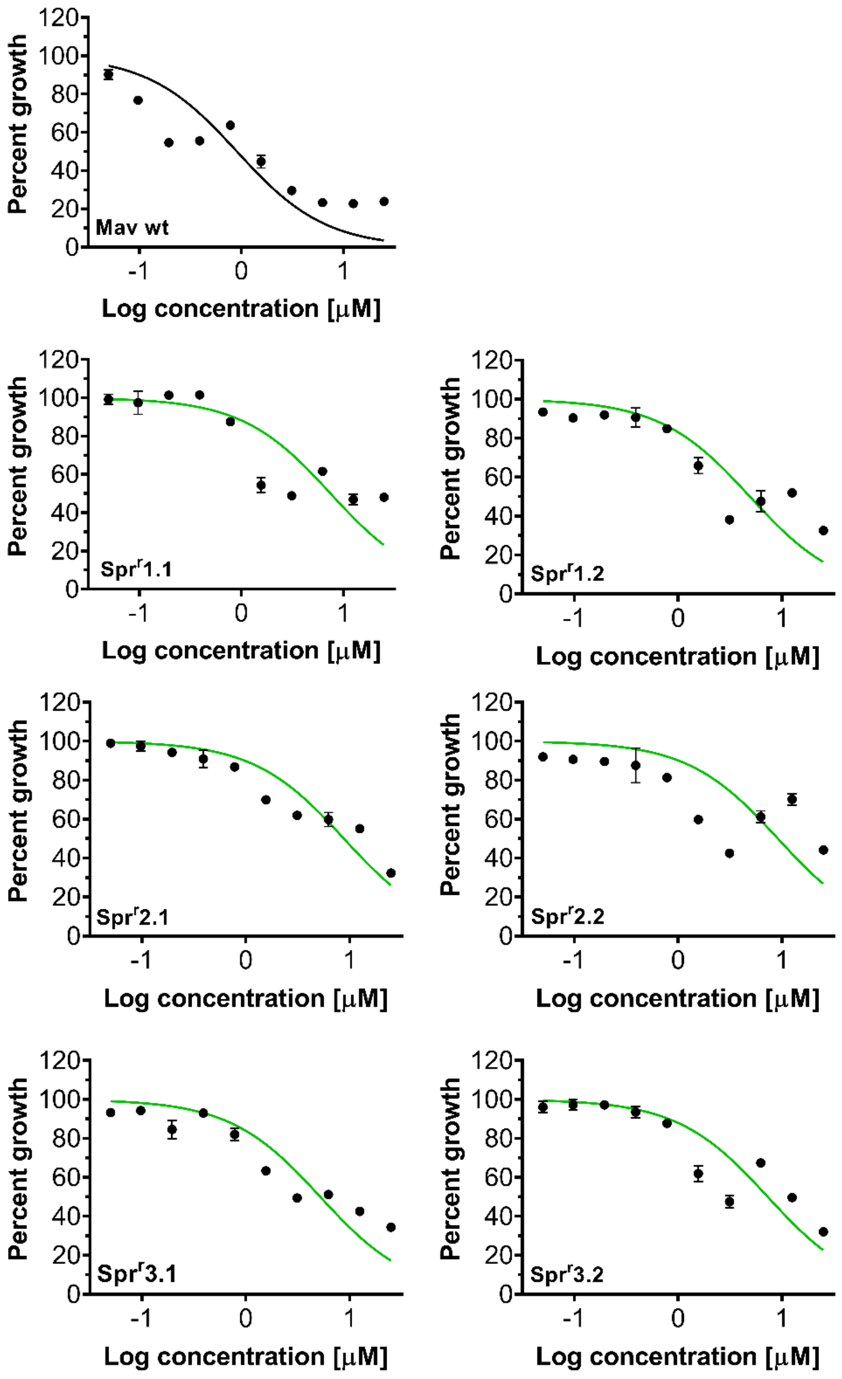

FIG. S1. SPR719 growth inhibition dose response curves of SPR719 resistant *M. avium* subsp. *hominissuis* 109. See Table 1 for strains, MICs and genotypes. The experiments were carried out three times independently and mean values with standard deviations are shown.

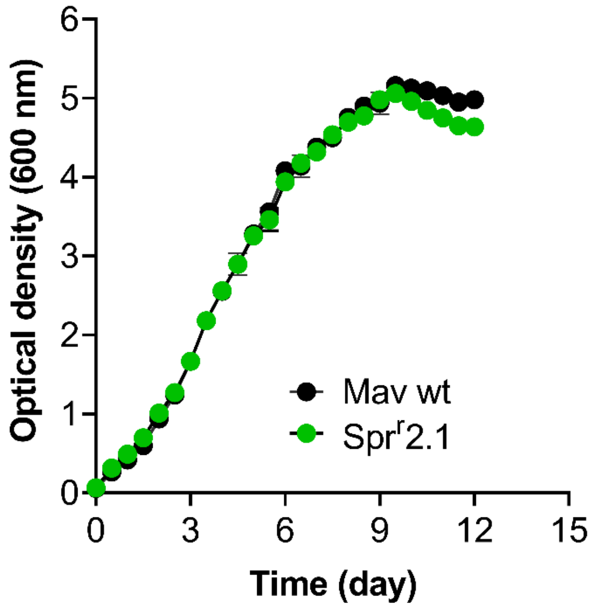

FIG. S2. *In vitro* growth curve of a representative SPR719 resistant strain versus parent wild type *M. avium* subsp. *hominissuis* 109. See Table 1 for strains, MICs and genotypes. The experiment was carried out three times independently and mean values with standard deviations are shown.

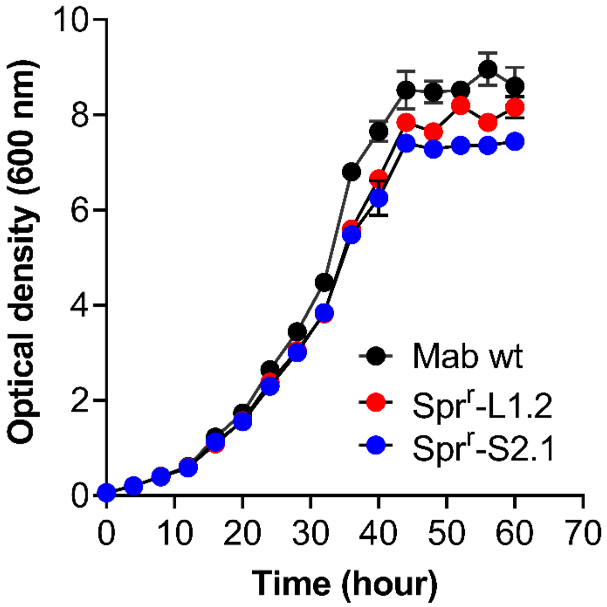

FIG. S3. *In vitro* growth curves of representative SPR719 resistant large (Spr^r-L1.2) and small (Spr^r-S2.1) colony morphotype strains versus parent wild type *M. abscessus* subsp. *abscessus* ATCC19977. See Table 2 for strains, MICs and genotypes. The experiment was carried out three times independently and mean values with standard deviations are shown.

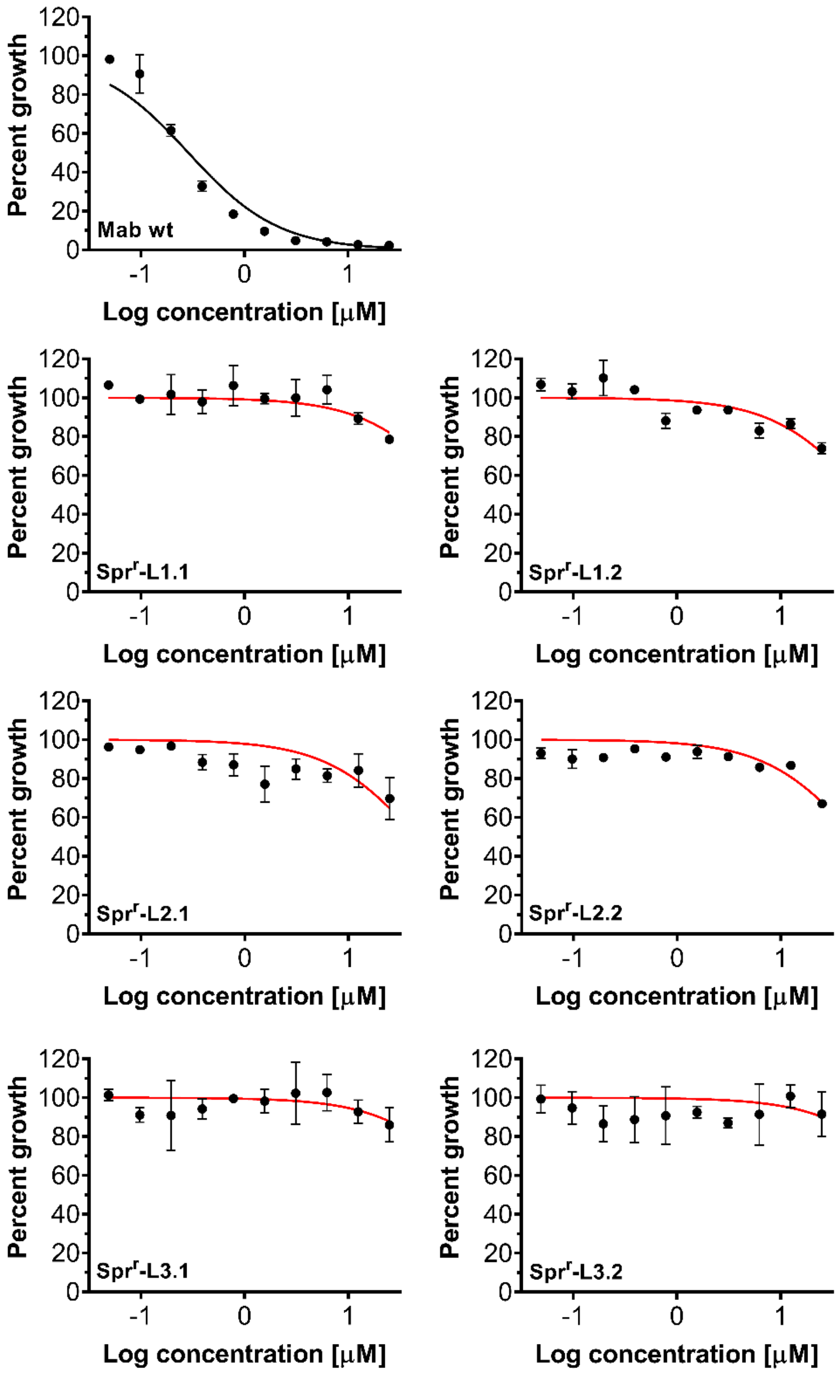

FIG. S4. SPR719 growth inhibition dose response curves for SPR719 resistant, large colony morphotype *M. abscessus* subsp. *abscessus* ATCC19977 strains. See Table 2 for strains, MICs and genotypes. The experiments were carried out three times independently and mean values with standard deviations are shown.

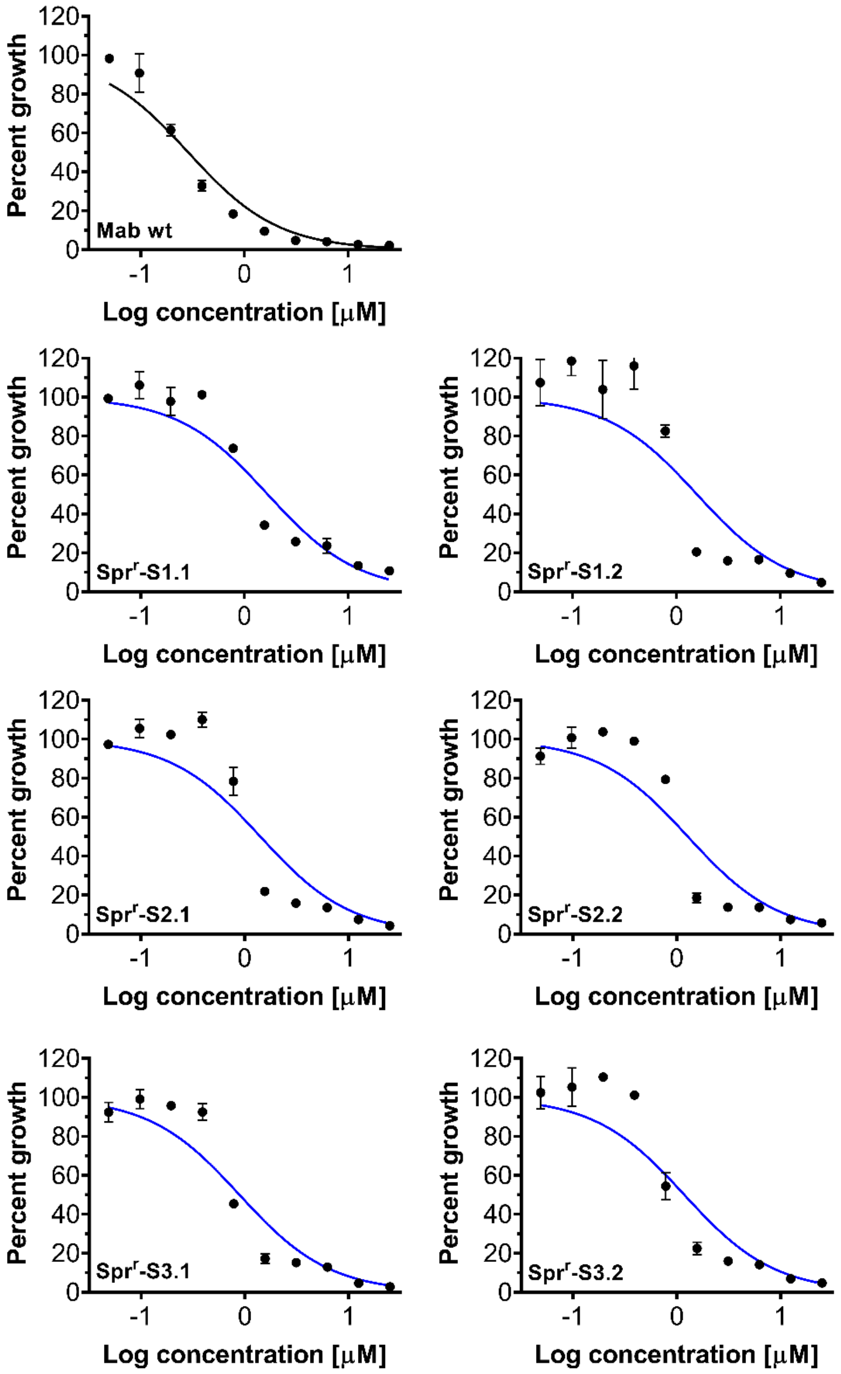

FIG. S5. SPR719 growth inhibition dose response curves for SPR719 resistant, small colony morphotype *M. abscessus* subsp. *abscessus* ATCC19977 strains. See Table 2 for strains, MICs and genotypes. The experiments were carried out three times independently and mean values with standard deviations are shown.
